# Nutritional and Metabolic Derangements in Pancreatic Cancer and Pancreatic Resection

**DOI:** 10.3390/nu9030243

**Published:** 2017-03-07

**Authors:** Taylor M. Gilliland, Nicole Villafane-Ferriol, Kevin P. Shah, Rohan M. Shah, Hop S. Tran Cao, Nader N. Massarweh, Eric J. Silberfein, Eugene A. Choi, Cary Hsu, Amy L. McElhany, Omar Barakat, William Fisher, George Van Buren

**Affiliations:** The Elkins Pancreas Center, Michael E. DeBakey Department of Surgery, and Dan L. Duncan Cancer Center, Baylor College of Medicine, Houston, TX 77030, USA; Taylor.Gilliland@bcm.edu (T.M.G.); Nicole.Villafane@bcm.edu (N.V.-F.); Kevin.Shah@bcm.edu (K.P.S.); Rohan.Shah@bcm.edu (R.M.S.); Hop.TranCao@bcm.edu (H.S.T.C.); Nader.Massarweh@bcm.edu (N.N.M.); ejs@bcm.edu (E.J.S.); Eugene.Choi@bcm.edu (E.A.C.); Cary.Hsu@bcm.edu (C.H.); Amy.McElhany@bcm.edu (A.L.M.); Omar.Barakat@bcm.edu (O.B.); wfisher@bcm.edu (W.F.)

**Keywords:** pancreas cancer, pancreas adenocarcinoma, nutrition, pancreatic exocrine insufficiency, diabetes mellitus, pancreaticoduodenectomy, distal pancreatectomy, malnutrition, pancreatogenic diabetes, enteral nutrition

## Abstract

Pancreatic cancer is an aggressive malignancy with a poor prognosis. The disease and its treatment can cause significant nutritional impairments that often adversely impact patient quality of life (QOL). The pancreas has both exocrine and endocrine functions and, in the setting of cancer, both systems may be affected. Pancreatic exocrine insufficiency (PEI) manifests as weight loss and steatorrhea, while endocrine insufficiency may result in diabetes mellitus. Surgical resection, a central component of pancreatic cancer treatment, may induce or exacerbate these dysfunctions. Nutritional and metabolic dysfunctions in patients with pancreatic cancer lack characterization, and few guidelines exist for nutritional support in patients after surgical resection. We reviewed publications from the past two decades (1995–2016) addressing the nutritional and metabolic status of patients with pancreatic cancer, grouping them into status at the time of diagnosis, status at the time of resection, and status of nutritional support throughout the diagnosis and treatment of pancreatic cancer. Here, we summarize the results of these investigations and evaluate the effectiveness of various types of nutritional support in patients after pancreatectomy for pancreatic adenocarcinoma (PDAC). We outline the following conservative perioperative strategies to optimize patient outcomes and guide the care of these patients: (1) patients with albumin < 2.5 mg/dL or weight loss > 10% should postpone surgery and begin aggressive nutrition supplementation; (2) patients with albumin < 3 mg/dL or weight loss between 5% and 10% should have nutrition supplementation prior to surgery; (3) enteral nutrition (EN) should be preferred as a nutritional intervention over total parenteral nutrition (TPN) postoperatively; and, (4) a multidisciplinary approach should be used to allow for early detection of symptoms of endocrine and exocrine pancreatic insufficiency alongside implementation of appropriate treatment to improve the patient’s quality of life.

## 1. Introduction

Pancreatic adenocarcinoma (PDAC) is a morbid disease. Outcomes have improved for many malignancies; however, PDAC continues to increase in incidence and mortality. The incidence of PDAC in the United States in 2016 is estimated to be over 53,000 cases. It has steadily increased by 1.2% each year, from 2000 to 2012. PDAC is expected to be the second leading cause of cancer-related mortality by 2030 [1,2].

PDAC has an indolent presentation, and there are currently no reliable forms of early detection. Most patients do not show clinical symptoms until the disease becomes locally advanced or metastatic. Less than 20% of patients are able to undergo potentially curative resection due to the late presentation of the disease [1]. Overall survival (OS) rates for all stages of PDAC are low, with 1- and 5-year rates of 29% and 7% respectively. In comparison, pancreatic neuroendocrine tumors (PNETs) have a less lethal 53% 5-year OS for all stages (including functional and non-functional tumors); however, they can be quite morbid in presentation [1].

The pancreas has both endocrine and exocrine functions—the endocrine pancreas regulates metabolism in the body through the production of insulin and glucagon, while the exocrine pancreas primarily produces the enzymes necessary for digestion [3]. In the setting of cancer, both endocrine and exocrine functions are affected. Pancreatic exocrine insufficiency (PEI) manifests as weight loss and steatorrhea, while endocrine insufficiency may result in diabetes mellitus. Although both endocrine and exocrine cancers of the pancreas can arise, the prevalence of exocrine pancreatic cancer (95%) is much higher than that of endocrine pancreatic cancer, i.e., PNETs (5%) [1].

The pancreas is intimately involved in the metabolism of food and nutrients through its production and secretion of enzymes and hormones. Pancreas cancer causes aberrations which result in the distinct symptoms of malnutrition and altered glucose homeostasis. Malnutrition—a condition in which patient caloric intake fails to meet metabolic demands—is common in patients suffering from pancreatic cancer. This may result in a catabolic state due to a combination of inadequate intake of nutrients and a pathologic process of increased nutrient consumption as a result of tumor cytokine release. Studies have shown that many pancreatic cancer patients suffer from malnutrition due to physiologically-induced anorexia, malabsorption, and increased caloric requirements, resulting in weight loss. Over 80% of pancreatic cancer patients report weight loss at the time of diagnosis and over a third of these patients have lost greater than 10% of their initial body weight [4,5,6,7,8]. Many of these patients then experience cachexia, a severe condition involving pathological weight loss due to the wasting of skeletal muscle and adipose tissue [9]. Ultimately, pancreatic cancer patients with malnutrition or cachexia experience a lower quality of life, increased morbidity and mortality, longer hospital stays, and a reduced response to treatment [8,10,11,12].

The purpose of this literature review is to compare the nutritional and metabolic status of patients with pancreatic cancer at the time of diagnosis and at the time of resection, and to evaluate the effectiveness of various types of nutritional support in patients after pancreatectomy for PDAC.

## 2. Materials and Methods

### 2.1. Search Strategy

A search of the PubMed database between the years of 1995–2016 was performed to identify studies addressing the nutritional state in patients with pancreatic cancer, as well as the nutritional interventions before and after a pancreatic resection. We also reviewed the references of the studies and reviews included. Search terms included ‘pancreas cancer’, ‘pancreatic adenocarcinoma’, ‘Whipple’, ‘pancreaticoduodenectomy’, ‘distal pancreatectomy’, ‘nutrition’, ‘malnutrition’, ‘weight loss’, ‘outcomes’, ‘metabolism’, ‘exocrine insufficiency’, ‘pancreatic enzymes’, ‘diabetes mellitus’, ‘pancreatogenic diabetes’, ‘enteral nutrition’, and ‘parenteral nutrition’.

### 2.2. Selection Criteria and Data Extraction

A review of randomized and non-randomized studies was conducted. The manuscripts were separated into three categories for further analysis: presenting symptoms upon the diagnosis of pancreatic cancer, symptoms after surgical resection, and nutritional support throughout the diagnosis and treatment of pancreatic cancer. Studies that qualified for more than one category were placed in the category of best fit to avoid duplication and were marked for reevaluation upon a synthesis of the review. Inclusion criteria for the review encompassed primary data-containing manuscripts regarding patient nutritional status before or after surgery, malignant pancreatic disease, a description of markers of malnutrition and exocrine and/or endocrine function, and data on intervention with total parental or enteral nutrition. We excluded case reports (4), editorials (3), manuscripts that focused primarily on chemotherapy or radiation (3), and manuscripts that focused on other gastrointestinal malignancies besides pancreatic cancer (2).

### 2.3. Quality Assessment

Two investigators (T.M.G. and R.M.S.) independently reviewed all titles and abstracts for inclusion. Full texts of the eligible studies were retrieved and re-evaluated for compliance with the inclusion criteria. The information extracted included the author, publication year, study design, statistical analysis, patient baseline characteristics, markers of nutrition, vitamin deficiencies, pancreatic function, evaluation before and/or after pancreaticoduodentomy or distal pancreatectomy, type of nutritional intervention, complications, and overall morbidity and mortality.

## 3. Results

### 3.1. Literature Research

The search produced a total of 4989 articles on PubMed. After excluding duplicate records of the same article from separate combination searches of the keywords described, 600 article titles were able to be readily reviewed on PubMed. A total of 63 of the articles were readily accessible with full text. We included 63 manuscripts focusing on the nutritional status of patients undergoing pancreaticoduodenectomy (PD) and distal pancreatectomy (DP), and those comparing enteral or parental nutritional interventions prior to or after surgery for PDAC in our analysis. Based on the exclusion criteria, an additional 12 articles were excluded upon a second review.

### 3.2. Study Characteristics

**Figure 1:**
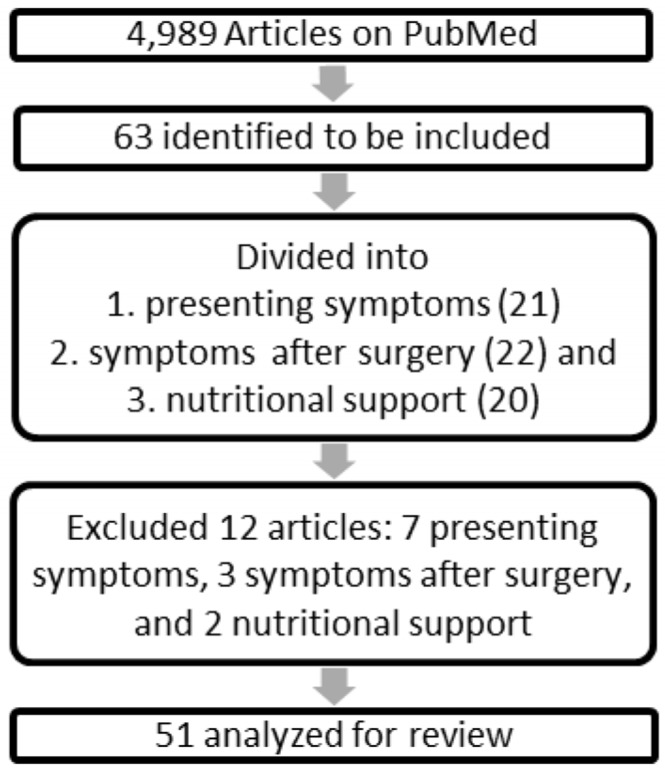
Literature search and study.

A total of 51 articles were utilized for the review, with 14 addressing malnutrition and pancreatic dysfunction at pancreatic cancer diagnosis [13,14,15,16,17,18,19,20,21,22,23,24,25,26]; 19 addressing pancreatic dysfunction after surgery [27,28,29,30,31,32,33,34,35,36,37,38,39,40,41,42,43,44,45], including 10 which also discussed preoperative dysfunction and nutrition [27,30,31,32,33,35,37,39,40,41]; and 18 addressing nutritional intervention [11,46,47,48,49,50,51,52,53,54,55,56,57,58,59,60,61,62] (Figure 1).

### 3.3. Nutritional Status at Presentation

Weight loss is common at pancreatic cancer diagnosis and is a good marker of malnutrition. A percentage of weight loss greater than 5% is associated with a greater surgical site infection (SSI) rates and longer hospital stay. Unintentional weight loss, with an average of 5%, is predictive of a higher Malnutrition Universal Screen Tool (MUST) score, which is associated with increased morbidity and mortality. Hypoalbuminemia, defined in studies as albumin levels between 2.1 and 3.5 mg/dL, is predictive of greater postoperative complications. The Prognostic Nutrition Index (PNI) incorporates albumin into its risk assessment and a score of less than 45 is predictive of postoperative complications. Contributing to weight loss and malnutrition in pancreatic cancer is PEI, which is diagnosed by the presence of fecal elastase. A symptom of PEI is steatorrhea, which is subjective in nature and is a poor measure of adequate enzyme replacement. Another metabolic imbalance, prevalent due to the disease process, is endocrine dysfunction. Long standing diabetes is a risk factor for PDAC and new onset diabetes within two years of diagnosis is a recognized phenomenon. New-onset diabetes mellitus (NODM) is pancreatogenic or Type 3c in nature. It is associated to deficiencies in pancreatic polypeptide and incretins.

### 3.4. Nutritional Status at Time of Resection

Postoperatively, patients typically recover serum markers after three months and relative body weight after six months. However, patients may continue to have fat-soluble vitamin and mineral deficiencies. Classic PD presents an especially increased risk of vitamin B12 and zinc deficiency. Furthermore, those with PD more commonly have exocrine insufficiency postoperatively, and require enzyme replacement therapy. Currently, there is no objective measurement for adequate enzyme supplementation. The reported incidence range of diabetes mellitus (DM) after resection is less consistent, which may be correlated to the percent volume of resected pancreas. Chronic pancreatitis and preexisting DM are risk factors for continued DM after a pancreatic resection, whereas the resolution of DM occurs more often after PD. It is unclear whether the immediate postoperative endocrine function is predictive of survival one year after surgery.

### 3.5. Nutritional Support

Enteral nutrition (EN) support for pancreatic cancer patients undergoing PD is preferred over total parenteral nutrition (TPN). EN displays better substrate utilization, maintains gastrointestinal integrity, and reduces complications, while improving nutritional status. The utility of immune-enhancing EN, which contains fatty acids and vitamins, may reduce morbidity and mortality. The combined use of EN and TPN does not significantly impact morbidity in patients postoperatively. TPN still plays a role in providing nutritional support and may be indicated in those at a dangerous risk of starvation or severe cachexia. Eicosapentaenoic acid (EPA) supplementation may help patients gain weight. The use of postoperative feeding jejunostomy tubes (FJT), nasojejunal early enteral nutrition (NJEEN), or naso-jejunal tube (NJT) in patients undergoing PD, is a decision made on a case-by-case basis. Hypoalbuminemia has been indicated as a risk factor for FJT complications, and NJEEN has been associated with more postoperative pancreatic fistulas compared to TPN. NJT is similar to oral feedings in complication rates.

## 4. Discussion

### 4.1. Nutritional Deficiencies in Pancreatic Adenocarcinoma Presentation

#### 4.1.1. Weight Loss & Biochemical Markers

Cancer-associated weight loss is a well-established phenomenon. A total of 80% of patients with pancreatic head cancer present with weight loss at diagnosis [16,31,63], with up to 40% having a greater than 10% weight loss within six months of diagnosis [16]. Weight loss is a recognized marker of malnutrition and is associated with a poor prognosis. Several case-control and cohort studies have evaluated the utility of nutritional risk scores, as well as the degree of weight loss, as factors in determining malnutrition and postoperative morbidity and mortality. In Kanda et al., the percent of weight loss was a risk factor for higher degrees of malnutrition, but it was not associated with a change in overall survival or stage of disease [16,18]. Body Mass Index (BMI) has been consistently proven to be a poor tool for assessing the nutritional status and disease prognosis in pancreatic cancer patients [18,20,27]. It often underestimates the degree of nutritional dysfunction. Patients with higher BMIs experienced greater weight loss than patients with underweight BMIs prior to cancer diagnosis [16]. Moreover, a low or underweight BMI did not correlate to a worse overall survival rate in pancreatic cancer patients [18].

The PNI and MUST are nutritional risk scores that are routinely used to assess the risk of malnutrition, in both surgical and medical patients. PNI incorporates albumin and the peripheral blood lymphocyte count for identifying clinically relevant malnutrition and predicting short- and long-term postoperative outcomes. In a study of pancreatic cancer patients by Kanda et al., a low preoperative PNI score was associated with worse perioperative and greater postoperative complications, including the development of pancreatic fistulas, when compared to patients with a normal preoperative PNI. However, the PNI score has a limited utility in predicting long-term survival [18]. The MUST score incorporates unplanned weight loss, BMI, and acute disease effect (actively ill), to stratify hospitalized or acutely ill patients into high and low risk groups for malnutrition [64]. In a study by LaTorre et al., pancreatic cancer patients with high MUST scores had an increased 30-day morbidity and mortality, with a 50% higher 30-day morbidity than patients with low MUST scores [27]. In a separate study by Loh et al., unintentional weight loss in solid tumor malignancies, and not BMI, was an independent risk factor for patients with a high MUST score, who had an average of 5% weight loss [20]. Hypoalbuminemia, defined as a serum albumin level < 2.1 mg/dL, is a common surrogate marker of malnutrition and is independently associated with greater postoperative complications, such as surgical site infections (SSI) and postoperative morbidity and mortality [18,21,22,27]. In a prospective observational study of patients who underwent various surgeries, Gibbs et al. showed that the postoperative 30-day mortality rate changed from 1% with albumin levels > 4.6 mg/dL, to 28% with albumin levels < 2.1 mg/dL. In other studies, hypoalbuminemia, defined as either an albumin < 3.0 or <3.5 mg/dL, was used as a component of perioperative predictive scoring models that assess frailty and fitness to predict outcomes for pancreatic cancer and pancreatic surgery [23,24,25]. Complicating the use of albumin as a marker is the fact that serum albumin can be altered by acute inflammatory states, including recent trauma, surgery, or infections. One of the challenges of implementing any of the various scoring systems and perioperative models is that albumin and nutritional factors are often a component of a much broader picture. Furthermore, the values that were used as a cut-off, vary from study to study.

#### 4.1.2. Pancreatic Exocrine Insufficiency (PEI)

PEI contributes to malnutrition and weight loss in pancreatic cancer. PEI results in malabsorption, due to the inability of the pancreas to generate and secrete digestive enzymes into the duodenum secondary to tumor obstruction and fibrosis of the pancreatic ducts [13,63]. In Sikkens et al., PEI was present in over half of PDAC patients at the time of diagnosis and is best diagnosed by the measurement of fecal elastase [13]. Symptoms, such as steatorrhea, were not always apparent with enzyme insufficiency. In Hakert et al., PEI was thought to manifest when the pancreatic lipase was 5%–10% of the normal output [63]. This creates a conundrum when patients are given pancreatic enzyme supplementation to combat PEI. Supplementation can improve steatorrhea as a symptom, but does not necessarily indicate the proper absorption of nutrients, or ultimately improve the nutritional status of patients [31,65].

#### 4.1.3. Pancreatic Endocrine Insufficiency

Additionally, altered endocrine homeostasis is often present at pancreatic cancer diagnosis. DM occurs more frequently in patients with pancreatic cancer than in the general population [15]. Approximately 50% of patients are diabetic or insulin deficient at the time of cancer diagnosis; of these, half are diagnosed within three years of diabetes diagnosis [14,17,19,66]. Additionally, long standing DM of greater than 20 years has been shown to increase the risk of pancreatic cancer by 1.5-fold [19]. Despite these associations, the relationship between DM and pancreatic cancer is complex and not fully elucidated.

NODM, defined as DM diagnosed within 12 months, is associated to a two-fold higher incidence of pancreatic cancer [67]. NODM in pancreatic cancer patients was associated with a younger age at cancer diagnosis (between 45–49 years old) and a >10% weight loss at presentation [16,26]. Those who were diagnosed with DM follow other conventional risk factors, including a higher BMI and family history of DM [19,26]. Roeyen et al. and Pannala et al. showed higher incidences of NODM, at about 75%, in the two years prior to pancreatic cancer diagnosis [15,19]. However, these results did not capture all NODM, since approximately 34% were identified as undiagnosed DM in the study by Roeyen et al. [15]. Furthermore, 35% of patients had decreased endocrine function through impaired fasting glucose or glucose intolerance [15].

Type 3c DM, also known as pancreatogenic diabetes, originates as a consequence of pancreatic disease, secondary to a deficiency in the nutrient-stimulated release of pancreatic polypeptide, which regulates endocrine and exocrine function. It occurs in the absence of autoantibodies [26,66,68]. Of all patients with DM, 8% have pancreatogenic diabetes. A total of 75% of patients with this form of diabetes differ from the more common type 2 diabetes. Type 2 diabetes is characterized by decreased insulin sensitivity. In T3cDM, the endocrinopathy is very complex, since it is affected by additional comorbidities, such as maldigestion and concomitant qualitative malnutrition. Associated pathological conditions, such as exocrine insufficiency, the lack of fat-soluble vitamins (especially vitamin D), impairment of fat hydrolysis, and impairment of incretin secretion, are frequently found in T3cDM. Incretins are a group of metabolic hormones that stimulate insulin and inhibit glucagon [26,66]. T3cDM is usually associated with a history of chronic pancreatitis—a significant risk factor for PDAC. The stage and location of the tumor has not been shown to influence the presence of DM, while the frequency of Type 3c diabetes in the setting of PDAC is still being defined in the literature [19].

### 4.2. Post-surgical Changes in Nutritional Status

Pancreatic surgery significantly affects pancreatic function and patients’ nutritional status. PD may be complicated by pancreatic fistula, delayed gastric emptying, dumping syndrome, weight loss, DM, and nutritional deficiencies. Nutritional status decreases with surgery, but often recovers to preoperative levels, despite an altered absorption of nutrients.

#### 4.2.1. Biochemical Markers

In a prospective study of predominately PD patients, the relative body weight and skinfold thickness of the triceps decreased after surgery, but returned to preoperative levels within six months of follow-up [30]. Comparatively, biochemical markers of nutrition, such as transferrin, albumin, and total protein, were lowest at discharge, but recovered within three months of follow-up [30]. This suggests that physical markers of nutritional status may take longer to recover than biochemical markers. It is unclear whether patients with marked preoperative malnutrition recovered their lost weight and nutritional markers as successfully in the postoperative period [30].

#### 4.2.2. Vitamin Deficiencies

Vitamin deficiencies occur after PD, due to resected bowel, altered gastrointestinal anatomy, and insufficient levels of pancreatic enzymes. In classic PD, the gastric antrum is resected, and there is a subsequent loss of intrinsic factor, which is necessary for B12 absorption. Patients are at high risk of vitamin B12 deficiency and often require monthly injections [69]. Diminished pancreatic enzymes increase the risk of fat-soluble vitamin deficiencies, especially with severe PEI [69]. Vitamin deficiencies are typically subclinical and require testing for vitamin A, E, or 25-OH-vitamin D3 levels [68,69]. The resection of the duodenum during the PD may place patients at risk of iron and mineral deficiencies [44]. Zinc deficiency was reported in up to 68% of pancreas resection patients, predominately after PD, although most were asymptomatic [34]. In contrast, Armstrong et al. did not identify zinc deficiency in classic PD patients [44] Suggested risk factors for zinc deficiency include standard PD (vs. pylorus-preserving PD), PEI, and a low pancreatic duct to parenchyma ratio [34]. Further research is needed to evaluate the risk and significance of these nutritional deficiencies in pancreatic head versus tail resection.

#### 4.2.3. Exocrine Insufficiency

PEI risk may differ, depending on the type of surgical resection, which is supported by the underlying pathophysiology. PD requires a parenchymal resection and extended lymphadenectomy, with a circumferential dissection of the nerve plexi and interstitial cells of Cajal. The dissection of the nerve plexi and interstitial cells of Cajal can potentially result in tonic inhibitory effects of the sympathetic nerves around the superior mesenteric artery (SMA), causing severe diarrhea and malnutrition [63,70]. Exocrine insufficiency may increase by 38% after surgery [31] and over half of patients require enzyme supplementation [28,42,43]. Fecal elastase levels will not recover after surgery, but steatorrhea typically resolves with adequate enzyme supplementation [30,42]. Since fecal elastase is the measure of a specific pancreatic enzyme, it should not recover with synthetic supplementation. Another tool is necessary to assess malabsorption and adequate enzyme supplementation, such as fecal fat. Unfortunately, approximately half of patients do not take adequate enzyme supplementation, restrict their fat intake, and do not consult with a dietician [28].

Steatorrhea is 40% less common after DP than after proximal resections [29,30]. This may be due to the preservation of the celiac and SMA plexus, and bilateral ganglions [63,70]. However, these studies do not evaluate preoperative steatorrhea, or differences in benign lesions, malignant lesions, and tumor type. This can be significant, since the most common indication for PD is PDAC of the head, compared to PNET in DP. As for the tumor type, PDAC likely results in a higher risk of PEI than ampullary adenocarcinomas [43].

#### 4.2.4. Endocrine Insufficiency

A resection of pancreatic parenchyma decreases the quantity of beta cells that produce the insulin necessary for glucose homeostasis. The incidence of DM after a surgical resection is still being defined. Experimental models have suggested fasting blood glucose impairment when >50% of beta cells are removed, while retrospective studies have shown that resection of 25%–44% of the pancreatic volume during DP can influence the development of such impairment [32,36,38,45]. However, You et al. found that the volume of the pancreas resected is not associated with changes in glucose metabolism, newly diagnosed DM, preoperative diabetes, or atrophic or hypertrophic changes to the pancreas [37]. Similarly, endocrine function after surgical resection of benign and malignant tumors can be unpredictable and has been shown to improve, worsen, or remain unchanged [29,30,32,35,42]. Studies grouping PD and DP showed that 17%–24% of patients are diagnosed with new DM after surgical resection [30,39]. New DM after PD ranged from 15% to 41% [29,37,40,42,43], and after DP, ranged from 8% to 54% [29,35,36,40]. Within these series, there was significant variability in the location of the tumor, tissue diagnosis, and presence of chronic pancreatitis, which confounds the data. Multivariate analysis in one study by Burkhart et al. demonstrated that newly diagnosed DM after DP, is significantly greater than after PD. Risk factors for new onset DM after surgery, included chronic pancreatitis and previous glucose intolerance [35,36,38,40]. In studies where preexisting DM worsened after a pancreatic resection, there was wide variability in PD and DP [35,37,40]. Chronic pancreatitis [30,41] and DP [30] correlated with exacerbation of preexisting DM.

Following surgery, DM or endocrine dysfunction may resolve, and the predictive value of immediate postoperative results is unclear. In Kang et al., the endocrine function at one week was predictive of the function at 12 months. In contrast, Park et al. demonstrated a transient rise in endocrine dysfunction markers which resolved by three months, and a 12% decrease in DM from six to 12 months. DM resolution occurred in 20%–57% of patients after PD [19,32,41], and 13% of patients after DP [33]. Conditions that prevented the resolution of DM after surgical resection included chronic pancreatitis, long standing DM, insulin use, previous glucose intolerance, and malignancy [19,30,32,39,41].

### 4.3. Nutritional Support

Nutritional interventions play a pivotal role in the successful management of patients after pancreatectomy. Malnutrition has been associated with an increase in the length of the hospital stay and a greater risk of morbidity and mortality, while reducing response to treatment and quality of life [11]. As mentioned earlier, malnutrition is broadly defined as a condition in which the body is in a catabolic state, due to an inadequate intake of nutrients or a pathologic process inhibiting the utilization of nutrients in the setting of adequate intake. However, discreet cut-offs for serum nutritional markers, such as albumin and prealbulmin, often do not encompass the full spectrum of patients who fall into this category. While the approaches are still being refined, a growing body of literature on this topic has yielded postoperative strategies to optimize patient outcomes.

#### 4.3.1. Total Parenteral Nutrition vs. Enteral Nutrition

Historically, total parenteral nutrition (TPN) was the first line nutritional intervention postoperatively for gastrointestinal cancer surgeries due to the long held belief that critically injured patients are unable to tolerate enteral feeding from paralytic ileus [51]. Today, enteral nutrition (EN) is overwhelmingly preferred and should be the first line method of feeding when possible. Enteral feeding displayed better substrate utilization, prevented mucosal atrophy, and maintained gastrointestinal integrity and immunocompetence, while improving protein kinetics [51,70]. EN has also been shown to reduce complications, hospital stay, and chemotherapy toxicity, while increasing energy intake and nutritional status [58]. Randomized control trials by Park et al. and Liu et al. on postoperative nutritional support involving PD patients showed the superiority of EN compared to TPN [52,53]. TPN has been associated with an increased rate of complications, longer duration to first bowel movement, and longer time until the resumption of a normal diet, when compared to EN [52,53,56].

#### 4.3.2. Immunomodulating Enteral Nutrition

Throughout the years, various clinicians have added macromolecules and other supplements to the EN formulas, with variable results. The studies by Park et al. and Liu et al. also show that immunomodulating EN, which has additional amino acids, vitamins, fatty acids, and nucleotides, was associated with lower postoperative complications, a shorter length of stay, and lower mortality and morbidity, when compared to standard EN and TPN [52,53]. Previous studies have shown mixed results with an immune-enhancing formula (IEF) [54,55]. Daly and others demonstrated a decrease in the incidence of infections and wound-related complications, while Kenlar et al. reported no difference in the number of patients with infections and no increase in the length of the hospital stay. Karagianni et al. and others have more recently shown that EN, immuno-enriched with arginine and omega-3-fatty acids, results in lower postoperative infections and a shorter hospital stay, when compared to that in standard EN and TPN [70]. These findings are further validated by a randomized control trial by Klek et al., which reported that surgical site infections, bacteremia, morbidity and mortality, and length of hospital stay were greater in standard EN than with immunomodulating EN with arginine, glutamine, omega-3-fatty acids, vitamins C and E, and nucleotides [47]. Medium chain fatty acids and protein-enriched EN have been shown to improve protein and prealbumin plasma levels, while reducing length of stay, when compared to isocaloric protein-enriched EN [70]. Clinicians have also tried a dual EN + TPN strategy to optimize postoperative nutritional status. Nagata et al. reported that patients who received EN with TPN, had a significantly higher rate of duration and discontinuance of enteral feeding, compared to those who just received EN [50]. There were no differences in postoperative morbidity, catheter-related infections, percent of weight loss, and postoperative length of stay.

#### 4.3.3. Total Parenteral Nutrition

TPN may have some role in preventing and treating severe cachexia [7,56,57]. Studies led by Pelzer et al. and Vashi et al. showed an improvement in the quality of life and Subjective Global Assessment (SGA) after three months of home TPN, as well as an overall increase in BMI, improved median phase angle, and decreased extracellular mass [56,57]. A phase II study also demonstrated the benefit of TPN in 32 outpatients with advanced cancer and progressive cachexia [70]. TPN has shown the ability to overcome a postoperative stress response by increasing protein synthesis and immune function, resulting in a decrease in postoperative infectious complications. Thus, TPN may be indicated for patients who are at a dangerously elevated risk of death from starvation and cannot consume through enteral means.

#### 4.3.4. Eicosapentaenoic Acid Supplementation

The use of eicosapentaenoic acid (EPA) supplementation as part of postoperative nutritional management after PD has increased among clinicians, although the benefits have not been well established. Wigmore et al. found a significant median weight gain of 0.5 kg after one month of EPA supplementation, that persists throughout the 12-week study period [58]. However, the total body water percentage, acute phase protein response, nutritional intake, and performance status for patients was unchanged in patients receiving EPA. A series of studies conducted by Barber and co-authors found that EPA has an anabolic effect, resulting in an increase in weight and fasting insulin, and a decrease in resting energy expenditure (REE) [59,62]. EPA may also improve postoperative liver and pancreas function in patients receiving TPN [60]. Further controlled studies are necessary in order to fully understand the potential benefits of EPA as a postoperative nutritional supplement.

#### 4.3.5. Use of Feeding Tubes Postoperatively

It is also important to consider the patient’s preoperative nutritional state when evaluating mechanisms for delivering postoperative nutrition. While feeding jejunostomy tubes (FJT) for patients undergoing PD have been associated with increased postoperative complications, the practice remains common [48]. Nussbaum et al. and others have attempted to better characterize the outcomes and morbidity associated with FJT. The authors identified preoperative hypoalbuminemia as the only independent predictor of FJT complications [48]. Patients with FJT were more likely to be on TPN while in the hospital, require TPN at discharge, and have higher rates of readmission and reoperation. As such, the criteria for FJT placement must be judiciously standardized, to ensure that FJTs are absolutely necessary. Furthermore, the use of nasojejunal early enteral nutrition (NJEEN) in patients undergoing pancreatectomy, remains a controversial topic within the field. In a recent multicenter randomized control trial, Perinel and others compared postoperative complication rates between patients receiving NJEEN with those receiving TPN [61]. Results showed that NJEEN was associated with a significantly higher frequency and severity of postoperative pancreatic fistula (POPF), but did not result in a significant difference in the incidence of postoperative hemorrhage, infection, grade of complications, and length of stay. In contrast, Gerritsen et al. found that early oral feeding after PD resulted in a decreased length of hospital stay and no differences in Clavien-Dindo Grade III or higher complications, delayed gastric emptying, postoperative hemorrhage, and mortality when compared to patients who received nasojejunal tube (NJT) feeding [49]. As such, introducing oral feeding earlier than previously estimated may reduce the hospitalization time with minimal secondary drawbacks in patient outcomes. Many current studies are evaluating the effect of early feeding as a component of Enhanced Recovery After Surgery (ERAS) protocols.

## 5. Conclusions

There are currently no widely accepted consensus guidelines available to guide the preoperative, operative, and postoperative care of patients with pancreatic cancer and benign pancreatic disease. We have attempted to distill several conservative recommendations to help guide the care of these patients. Hypoalbuminemia and >10% weight loss play a substantial role in determining patient outcomes. These markers are used in our institution’s clinical practice to provide guidelines for the preoperative nutritional optimization of pancreatic cancer patients to reduce surgical complications, morbidity, and mortality (Table 1). These practical guidelines apply to patients with albumin levels less than 2.1 to 3.5 mg/dL, which are associated with greater morbidity and mortality. Thus, it is presumed that patients with an albumin level of less than 2.5 mg/dL or weight loss > 10% require intense nutritional intervention to reduce postoperative complications. Surgery should be postponed until there is an improvement in serum markers and functional status. Those with moderately decreased albumin levels (<3.0 mg/dL) or weight loss > 5% should still receive some form of nutritional supplementation prior to surgery to avoid undesirable patient outcomes associated with an insufficient nutritional intervention. FJTs can help optimize nutrition, but it should be understood that there is an increased risk of complications when the albumin level is 3 mg/dL or less [48]. FJTs should be considered in patients without preoperative hypoalbuminemia who are having difficulty maintaining an optimal caloric intake or have an anticipated oral feeding difficulty postoperatively.

Other preoperative screening substances that can provide further insight into patient nutritional and metabolic status are prealbumin, C-reactive protein, fasting blood glucose, hemoglobin A 1 C, and fecal elastase. Such screening will aid in the beginning of appropriate enzyme supplementation, glucose management, and may predict worsening function after surgery. Similarly, screening postoperatively is important to prevent hypoglycemic events and malabsorption due to enzyme insufficiency. Endocrine function should be monitored frequently, up to a year after surgery, to assess the need for medication changes. A closer look at vitamins, particularly fat-soluble vitamins and vitamin B12, as well as micronutrients such as zinc and iron, should be considered to assess for postoperative malabsorption and the need for supplementation. Managing exocrine insufficiency is challenging and requires the assessment of multiple components, including fecal elastase, to establish the presence of exocrine insufficiency. Integrating diarrheal symptoms, fecal fat levels, and fat-soluble vitamin levels will guide appropriate synthetic enzyme supplementation.

The pancreas, as a dual exocrine and endocrine gland, plays a pivotal role in digestion, absorption of nutrients, and glucose metabolism. PDAC, regardless of the location within the gland, contributes to inflammation, desmoplastic changes, mass effect/obstruction of the pancreatic duct, and has been implicated in the secretion of diabetogenic substances [32,71]. Malnutrition is found in 50%–80% of gastrointestinal cancer patients and is associated with worse outcomes and longer hospital stays [72,73]. Preoperative nutritional optimization is paramount, not only to reduce post-operative complications, but also to reduce the additional burden of pancreatic parenchyma reduction and anatomical alterations, which affect the capacity to process and absorb nutrients. Postoperative morbidity and mortality following PD has significantly improved over the past several decades, however, a complication rate of approximately 40% persists on national level [74]. Endocrine and exocrine insufficiency after resection, combined with altered anatomy after gastrointestinal reconstruction, can result in the poor absorption of micronutrients and fat-soluble vitamins. The volume of the gland which is resected has been associated with the degree of functional impairment. PEI contributes to malnutrition and weight loss in both the preoperative and postoperative setting [13]. Glucose metabolism in pancreatic cancer and pancreatic resection patients remains a challenge. NODM after pancreatic resection has been described to range from 5%–42%, but evidence regarding the incidence of pancreatogenic diabetes after PD versus DP is currently limited [35].

Due to the risk of developing diabetes and malabsorption syndromes after a pancreatectomy, it is important to emphasize the need for a multidisciplinary approach to patients with PDAC. From the preoperative optimization with the use of immunonutrition to aid in the reduction of postoperative infectious complications, to postoperative early enteral feeding to promote gut integrity, surgeons, oncologists, and nutritionists must play an active role. The multidisciplinary approach enables the early detection of symptoms of pancreatic endocrine and exocrine insufficiency, and allows for the implementation of an appropriate treatment to improve the patient’s quality of life.

## Figures and Tables

**Table 1:** Perioperative Nutritional Recommendations.

Preoperative Nutrition Evaluation	Nutritional Intervention	Postoperative Nutritional Parameters to Follow
Albumin < 2.5 mg/dL or Weight Loss > 10%	Postpone surgery date. Begin aggressive nutrition supplementation Consider feeding jejunostomy tube (FJT) placement intraoperatively	Required: Hemoglobin A1c or Oral Glucose Tolerance Test (OGTT), Albumin, Fecal Elastase, and Vitamin B12 Recommended: Zinc, Iron, Vitamin A, Vitamin E, 25-OH-vitamin D3 and fecal fat
Weight Loss 5%–10% or Albumin < 3 mg/dL	Nutrition supplementation prior to surgery Consider FJT placement intraoperatively	

## Abbreviations

QOL	quality of life
PEI	pancreatic exocrine insufficiency
PNETs	pancreatic neuroendocrine tumors
PDAC	pancreatic adenocarcinoma
PD	pancreaticoduodenectomy
DP	distal pancreatectomy
TPN	total parenteral nutrition
DM	diabetes mellitus
PNI	Prognostic Nutrition Index
MUST	Malnutrition Universal Screen Tool
SSI	surgical site infections
NODM	New onset diabetes mellitus
3c DM	pancreatogenic diabetes
SMA	superior mesenteric artery
EN	enteral nutrition
BMI	body mass index
SGA	Subjective Global Assessment
EPA	eicosapentaenoic acid
REE	resting energy expenditure
FJT	feeding jejunostomy tubes
NJEEN	nasojejunal early enteral nutrition
NJT	naso-jejunal tube
POPF	postoperative pancreatic fistula

## References

[1] American Cancer Society: Cancer Facts & Figures 2016. http://www.cancer.org/content/dam/cancer-org/research/cancer-facts-and-statistics/annual-cancer-facts-and-figures/2016/cancer-facts-and-figures-2016.pdf.

[2] Rahib L., Smith B.D., Aizenberg R., Rosenzweig A.B., Fleshman J.M., Matrisian L.M. (2014). Projecting cancer incidence and deaths to 2030: The unexpected burden of thyroid, liver, and pancreas cancers in the united states. Cancer Res..

[3] Costanzo L.S. (2014). BRS Physiology. Physiology.

[4] Bye A., Jordhøy M.S., Skjegstad G., Ledsaak O., Iversen P.O., Hjermstad M.J. (2013). Symptoms in advanced pancreatic cancer are of importance for energy intake. Support. Care Cancer.

[5] Davidson W., Ash S., Capra S., Bauer J., Cancer Cachexia Study Group (2004). Weight stabilisation is associated with improved survival duration and quality of life in unresectable pancreatic cancer. Clin. Nutr..

[6] Ferrucci L.M., Bell D., Thornton J., Black G., McCorkle R., Heimburger D.C., Saif M.W. (2011). Nutritional status of patients with locally advanced pancreatic cancer: A pilot study. Support. Care Cancer.

[7] Richter E., Denecke A., Klapdor S., Klapdor R. (2012). Parenteral nutrition support for patients with pancreatic cancer—Improvement of the nutritional status and the therapeutic outcome. Anticancer Res..

[8] Gärtner S., Krüger J., Aghdassi A.A., Steveling A., Simon P., Lerch M.M., Mayerle J. (2016). Nutrition in Pancreatic Cancer: A Review. Gastrointest. Tumors.

[9] Mueller T.C., Burmeister M.A., Bachmann J., Martignoni M.E. (2014). Cachexia and pancreatic cancer: Are there treatment options?. World J. Gastroenterol..

[10] Fearon K., Baracos V. (2010). Cachexia in pancreatic cancer: New treatment options and measures of success. HPB.

[11] Kyle U.G., Pirlich M., Lochs H., Schuetz T., Pichard C. (2005). Increased length of hospital stay in underweight and overweight patients at hospital admission: A controlled population study. Clin. Nutr..

[12] Bachmann J., Heiligensetzer M., Krakowski-Roosen H., Büchler M.W., Friess H., Martignoni M.E. (2008). Cachexia worsens prognosis in patients with resectable pancreatic cancer. J. Gastrointest. Surg..

[13] Sikkens E.C.M., Cahen D.L., de Wit J., Looman C.W.N., van Eijck C., Bruno M.J. (2014). A prospective assessment of the natural course of the exocrine pancreatic function in patients with a pancreatic head tumor. J. Clin. Gastroenterol..

[14] Illés D., Terzin V., Holzinger G., Kosar K., Roka R., Zsori G., Abraham G., Czako L. (2016). New-onset type 2 diabetes mellitus—A high-risk group suitable for the screening of pancreatic cancer?. Pancreatology.

[15] Roeyen G., Jansen M., Chapelle T., Bracke B., Hartman V., Ysebaert D., De Block C. (2016). Diabetes mellitus and pre-diabetes are frequently undiagnosed and underreported in patients referred for pancreatic surgery. A prospective observational study. Pancreatology.

[16] Olson S.H., Xu Y., Herzog K., Saldia A., DeFilippis E.M., Li P., Allen P.J., O’Reilly E.M., Kurtz R.C. (2015). Weight Loss, Diabetes, Fatigue, and Depression Preceding Pancreatic Cancer. Pancreas.

[17] Gupta S., Vittinghoff E., Bertenthal D., Corley D., Shen H., Walter L.C., McQuaid K. (2006). New-Onset Diabetes and Pancreatic Cancer. Clin. Gastroenterol. Hepatol..

[18] Kanda M., Fujii T., Kodera Y., Nagai S., Takeda S., Nakao A. (2011). Nutritional predictors of postoperative outcome in pancreatic cancer. Br. J. Surg..

[19] Pannala R., Leirness J.B., Bamlet W.R., Basu A., Petersen G.M., Chari S.T. (2008). Prevalence and Clinical Profile of Pancreatic Cancer-Associated Diabetes Mellitus. Gastroenterology.

[20] Loh K.W., Vriens M.R., Gerritsen A., Borel Rinkes I.H.M., van Hillegersberg R., Schippers C., Steenhagen E., Ong T.A., Moy F.M., Molenaar I.Q. (2012). Unintentional weight loss is the most important indicator of malnutrition among surgical cancer patients. Neth J. Med..

[21] Billingsley K.G., Hur K., Henderson W.G., Daley J., Khuri S.F., Bell R.H. (2003). Outcome After Pancreaticoduodenectomy for Periampullary Cancer: An Analysis from the Veterans Affairs National Surgical Quality Improvement Program. J. Gastrointest. Surg..

[22] Gibbs J., Cull W., Henderson W., Daley J., Hur K., Khuri S.F. (1999). Preoperative Serum Albumin Level as a Predictor of Operative Mortality and Morbidity. Arch. Surg..

[23] Augustin T., Burstein M.D., Schneider E.B., Morris-Stiff G., Wey J., Chalikonda S., Walsh R.M. (2016). Frailty predicts risk of life-threatening complications and mortality after pancreatic resections. Surgery.

[24] Imaoka H., Mizuno N., Hara K., Hijioka S., Tajika M., Tanaka T., Ishihara M., Yogi T., Tsutsumi H., Fujiyoshi T. (2016). Evaluation of Modified Glasgow Prognostic Score for Pancreatic Cancer: A Retrospective Cohort Study. Pancreas.

[25] Lucas D.J., Schexneider K.I., Weiss M., Wolfgang C.L., Frank S.M., Hirose K., Ahuja N., Makary M., Cameron J.L., Pawlik T.M. (2014). Trends and Risk Factors for Transfusion in Hepatopancreatobiliary Surgery. J. Gastrointest. Surg..

[26] Hart P.A., Baichoo E., Bi Y., Hinton A., Kudva Y.C., Chari S.T. (2015). Pancreatic polypeptide response to a mixed meal is blunted in pancreatic head cancer associated with diabetes mellitus. Pancreatology.

[27] La Torre M., Ziparo V., Nigri G., Cavallini M., Balducci G., Ramacciato G. (2013). Malnutrition and pancreatic surgery: Prevalence and outcomes. J. Surg. Oncol..

[28] Sikkens E.C.M., Cahen D.L., van Eijck C., Kuipers E.J., Bruno M.J. (2012). The Daily Practice of Pancreatic Enzyme Replacement Therapy After Pancreatic Surgery: A Northern European Survey: Enzyme Replacement After Surgery. J. Gastrointest. Surg..

[29] Sabater L., Calvete J., Aparisi L., Canovas R., Munoz E., Anon R., Rosello S., Rodriguez E., Campus B., Alfonso R. (2009). Pancreatic and periampullary tumours: Morbidity, mortality, functional results, and long-term survival. Cirugía Española.

[30] Park J.W., Jang J.Y., Kim E.J., Kang M.J., Kwon W., Chang Y.R., Han I.W., Kim S.W. (2013). Effects of pancreatectomy on nutritional state, pancreatic function and quality of life. Br. J. Surg..

[31] Sikkens E.C.M., Cahen D.L., De Wit J., Looman C.W.N., Van Eijck C., Bruno M.J. (2014). Prospective assessment of the influence of pancreatic cancer resection on exocrine pancreatic function. Br. J. Surg..

[32] Kang J.S., Jang J.Y., Kang M.J., Kim E., Jung W., Chang J., Shin Y., Youngmin H., Kim S.W. (2015). Endocrine Function Impairment after Distal Pancreatectomy: Incidence and Related Factors. World J. Surg..

[33] Kang M.J., Jung H.S., Jang J.Y., Jung W., Chang J., Shin Y.C., Kim S.W. (2016). Metabolic effect of pancreatoduodenectomy: Resolution of diabetes mellitus after surgery. Pancreatology.

[34] Yu H.H., Yang T.M., Shan Y.S., Lin P.W. (2011). Zinc deficiency in patients undergoing pancreatoduodenectomy for periampullary tumors is associated with pancreatic exocrine insufficiency. World J. Surg..

[35] King J., Kazanjian K., Matsumoto J., Reber H.A., Yeh M.W., Hines O.J., Eibl G. (2008). Distal pancreatectomy: Incidence of postoperative diabetes. J. Gastrointest. Surg..

[36] Shirakawa S., Matsumoto I., Toyama H., Shinzeki M., Ajiki T., Fukumoto T., Ku Y. (2012). Pancreatic Volumetric Assessment as a Predictor of New-Onset Diabetes Following Distal Pancreatectomy. J. Gastrointest. Surg..

[37] You D.D., Choi S.H., Choi D.W., Heo J.S., Ho C.Y., Kim W.S. (2012). Long-term effects of pancreaticoduodenectomy on glucose metabolism. ANZ J. Surg..

[38] Ferrara M.J., Lohse C., Kudva Y.C., Farnell M.B., Que F.G., Reid-Lombardo K.M., Donohue J.H., Nagorney D.M., Chari S.T., Vege S.S. (2013). Immediate post-resection diabetes mellitus after pancreaticoduodenectomy: Incidence and risk factors. HPB.

[39] Hirata K., Nakata B., Amano R., Yamazoe S., Kimura K., Hirakawa K. (2014). Predictive Factors for Change of Diabetes Mellitus Status After Pancreatectomy in Preoperative Diabetic and Nondiabetic Patients. J. Gastrointest. Surg..

[40] Burkhart R.A., Gerber S.M., Tholey R.M., Lamb K.M., Somasundaram A., McIntyre C.A., Fradkin E.C., Ashok A.P., Felte R.F., Mehta J.M. (2015). Incidence and Severity of Pancreatogenic Diabetes After Pancreatic Resection. J. Gastrointest. Surg..

[41] Wu J.M., Ho T.W., Kuo T.C., Yang C.Y., Lai H.S., Chiang P.Y., Hsieh S.H., Lai F., Tien Y.W. (2015). Glycemic Change After Pancreaticoduodenectomy: A Population-Based Study. Medicine.

[42] Rault A., SaCunha A., Klopfenstein D., Larroude D., Dobo Epoy F.N., Collet D., Masson B. (2005). Pancreaticojejunal anastomosis is preferable to pancreaticogastrostomy after pancreaticoduodenectomy for longterm outcomes of pancreatic exocrine function. J. Am. Coll. Surg..

[43] Bock E.A., Hurtuk M.G., Shoup M., Aranha G.V. (2012). Late complications after pancreaticoduodenectomy with pancreaticogastrostomy. J. Gastrointest. Surg..

[44] Armstrong T., Strommer L., Ruiz-Jasbon F., Shek F.W., Harris S.F., Permert J., Johnson C.D. (2007). Pancreaticoduodenectomy for peri-ampullary neoplasia leads to specific micronutrient deficiencies. Pancreatology.

[45] Butler A.E., Janson J., Bonner-Weir S., Ritzel R., Rizza R.A., Butler P.C. (2003). Beta-cell deficit and increased beta-Cell apoptosis in humans with type 2 diabetes. Diabetes.

[46] Goonetilleke K.S., Siriwardena A.K. (2006). Systematic review of peri-operative nutritional supplementation in patients undergoing pancreaticoduodenectomy. JOP.

[47] Klek S., Sierzega M., Szybinski P., Szczepanek K., Scislo L., Walewska E., Kulig J. (2011). The immunomodulating enteral nutrition in malnourished surgical patients—A prospective, randomized, double-blind clinical trial. Clin. Nutr..

[48] Nussbaum D.P., Zani S., Penne K., Speicher P.J., Stinnett S.S., Clary B.M., White R.R., Tyler D.S., Blazer III D.G. (2014). Feeding Jejunostomy Tube Placement in Patients Undergoing Pancreaticoduodenectomy: An Ongoing Dilemma. J. Gastrointest. Surg..

[49] Gerritsen A., Wennink R.A.W., Besselink M.G.H., van Santvoort H.C., Tseng D.S.J., Steenhagen E., Borel Rinkes I.H.M., Molenaar I.Q. (2014). Early oral feeding after pancreatoduodenectomy enhances recovery without increasing morbidity. HPB.

[50] Nagata S., Fukuzawa K., Iwashita Y., Kabashima A., Kinoshita T., Wakasugi K., Maehara Y. (2009). Comparison of enteral nutrition with combined enteral and parenteral nutrition in post-pancreaticoduodenectomy patients: A pilot study. Nutr. J..

[51] Cooperman A., Chivati J., Chamberlain R. (2000). Nutritional and metabolic aspects of pancreatic cancer. Curr. Opin. Clin. Nutr. Metab. Care.

[52] Park J.S., Chung H.-K., Hwang H.K., Kim J.K., Yoon D.S. (2012). Postoperative Nutritional Effects of Early Enteral Feeding Compared with Total Parental Nutrition in Pancreaticoduodectomy Patients: A Prosepective, Randomized Study. J. Korean Med. Sci..

[53] Liu C., Du Z., Lou C., Wu C., Yuan Q., Wang J., Shu G., Wang Y. (2011). Enteral nutrition is superior to total parenteral nutrition for pancreatic cancer patients who underwent pancreaticoduodenectomy. Asia Pac. J. Clin. Nutr..

[54] Daly J., Weintraub F., Shou J., Rosato E., Lucia M. (1995). Enteral Nutrition During Multimodality Therapy in Upper Gastrointestinal Cancer Patients. Ann. Surg..

[55] Kenlar A., Swalis W., Dirscoll D. (1996). Early Enteral Feeding in Postsurgical Cancer Patients: Fish Oil Structed Lipid-Based Polymer Formula vs. Standard Polymerase Formula. Ann Surg..

[56] Pelzer U., Arnold D., Goevercin M., Stieler J., Doerken B., Riess H., Oettle H. (2010). Parenteral nutrition support for patients with pancreatic cancer. Results of a phase II study. BMC Cancer.

[57] Vashi P.G., Dahlk S., Popiel B., Lammersfeld C.A., Ireton-Jones C., Gupta D. (2014). A longitudinal study investigating quality of life and nutritional outcomes in advanced cancer patients receiving home parenteral nutrition. BMC Cancer.

[58] Wigmore S.J., Barber M.D., Ross J.A., Tisdale M.J., Fearon K.C.H. (2000). Effect of Oral Eicosapentaenoic Acid on Weight Loss in Patients With Pancreatic Cancer. Nutr. Cancer.

[59] Barber M.D., Fearon K.C.H., Tisdale M.J., McMillan D.C., Ross J.A. (2001). Effect of a Fish Oil-Enriched Nutritional Supplement on Metabolic Mediators in Patients With Pancreatic Cancer Cachexia. Nutr. Cancer.

[60] Heller A.R., Rössel T., Gottschlich B., Tiebel O., Menschikowski M., Litaz R.J., Zimmerman T., Koch T. (2004). Omega-3 fatty acids improve liver and pancreas function in postoperative cancer patients. Int. J. Cancer.

[61] Perinel J., Mariette C., Dousset B., Sielezneff I., Gainant A., Marbut J.Y., Bin-Dorel S., Bechwaty M.E., Delaunay D., Bernard L. (2016). Early Enteral Versus Total Parenteral Nutrition in Patients Undergoing Pancreaticoduodenectomy: A Randomized Multicenter Controlled Trial (Nutri-DPC). Ann. Surg..

[62] Barber M.D., McMillan D.C., Preston T., Ross J.A., Fearon K.C.H. (2000). Metabolic response to feeding in weight-losing pancreatic cancer patients and its modulation by a fish-oil-enriched nutritional supplement. Clin. Sci..

[63] Hackert T., Schütte K., Malfertheiner P. (2014). The pancreas: Causes for malabsorption. Visz. Gastrointest. Med. Surg..

[64] BAPEN (2011). “Malnutrition Universal Screening Tool” MAG the 5 “MUST” Steps e c Sc or Sc or Sc. http://www.bapen.org.uk/screening-and-must/must-calculator.

[65] Lindkvist B., Phillips M.E., Domínguez-Muñoz J.E. (2015). Clinical, anthropometric and laboratory nutritional markers of pancreatic exocrine insufficiency: Prevalence and diagnostic use. Pancreatology.

[66] Hillson R. (2016). Pancreatitis, pancreatic cancer, and diabetes. Pract. Diabetes.

[67] Giovannucci E., Michaud D. (2007). The Role of Obesity and Related Metabolic Disturbances in Cancers of the Colon, Prostate, and Pancreas. Gastroenterology.

[68] Timofte D., Livadariu R., Bintintan V., Diaconu C., Ionescu L., Sandbery A.A., Mariciuc D.C., Danila R. (2014). Metabolic disorders in patients operated for pancreatic cancer. Rev. Med. Chir. Soc. Med. Nat. Iasi.

[69] Keim V., Klar E., Poll M., Schoenberg M.H. (2009). Postoperative care following pancreatic surgery: Surveillance and treatment. Dtsch. Arzteblatt Int..

[70] Karagianni V.T., Papalois A.E., Triantafillidis J.K. (2012). Nutritional status and nutritional support before and after pancreatectomy for pancreatic cancer and chronic pancreatitis. Indian J. Surg. Oncol..

[71] Mori Y., Ohtsuka T., Tsutsumi K., Yasui T., Ueda J., Takahata S., Nakamura M., Tanaka M. (2012). Different incretin responses after pancreatoduodenectomy and distal pancreatectomy. Pancreas.

[72] Corish C.A., Kennedy N.P. (2000). Protein-energy undernutrition in hospital in-patients. Br. J. Nutr..

[73] Barker L.A., Gout B.S., Crowe T.C. (2011). Hospital malnutrition: Prevalence, identification and impact on patients and the healthcare system. Int. J. Environ. Res. Public Health.

[74] Gouma D.J., van Geenen R.C., van Gulik T.M., de Haan R.J., de Wit L.T., Busch O.R.C., Obertop H. (2000). Rates of complications and death after pancreaticoduodenectomy: Risk factors and the impact of hospital volume. Ann. Surg..

